# Community Awareness of Congenital Talipes Equinovarus (Clubfoot) in Makkah Region, Saudi Arabia: A Cross-Sectional Study

**DOI:** 10.7759/cureus.30602

**Published:** 2022-10-23

**Authors:** Mohammed A Alosaimi, Amjad M Jawhari, Omar A Amin, Essam S Alzahrani, Mohammed O Alomar, Mohammad T Nouri, Muhanna J Altalhi, Abdullah A Marzogi

**Affiliations:** 1 College of Medicine, Taif University, Taif, SAU; 2 Orthopedic Surgery, Alnoor Specialist Hospital, Makkah, SAU; 3 College of Medicine, King Saud University, Riyadh, SAU

**Keywords:** saudi arabia, makkah, ctev, clubfoot, knowledge, awareness

## Abstract

Background: Congenital talipes equinovarus (CTEV) or clubfoot is known as a deformity characterized by excessively turned-in feet and high medial longitudinal arches. It is one of the most common musculoskeletal abnormalities. It is estimated that approximately 20% of CTEV cases are caused by another congenital disease or syndromic condition.

Objectives: The aim of this study was to assess the knowledge about CTEV among the general population in the Makkah region of the kingdom.

Methods: This study was a community-based cross-sectional descriptive study carried on by an online questionnaire, previously validated in published studies, among residents in the Makkah region of Saudi Arabia who successfully fulfilled the inclusion and exclusion criteria.

Results: Out of the total number of respondents (n=1,987), gender was found to be significantly associated with awareness about CTEV (p-value=0.007) as females tend to have higher awareness levels than males. Having a child with CTEV was found to be significantly associated with awareness level (p-value˂0.001). In addition, university and secondary levels of education are more aware of CTEV than other levels of education (p-value=0.023).

Conclusion: According to the results, the lack of awareness campaigns may contribute to the low public awareness of CTEV. It is recommended that social media platforms and public campaigns be utilized to increase awareness of CTEV in key locations such as malls. These initiatives may motivate people to seek treatment for their disease as early as possible. In addition, early management of CTEV is less invasive and leads to better patient outcomes when followed up regularly.

## Introduction

Congenital talipes equinovarus (CTEV) also known as clubfoot, is one of the most common musculoskeletal deformities, which is characterized by excessively turned-in foot (equinovarus) and high medial longitudinal arch (cavus) [1]. There are approximately 80% of cases of isolated congenital talipes equinovarus (ICTEV), also known as idiopathic congenital talipes. Nonetheless, CTEV can be associated with other congenital diseases in 20% of cases, it can also be secondary or syndromic [2]. The cause of clubfoot is still not fully understood [3]. However, it is believed that it is a multifactorial disease in which both environmental and genetic factors play a role [4]. Previous studies reported a higher prevalence of clubfoot among male birth and first-born children [5-6].

The epidemiological studies on CTEV in the last 55 years showed a prevalence ranging from 0.5 to 2.0 cases per 1000 live births, which results in 7-43 cases of CTEV/year/million population, making it one of the most common congenital malformations in the pediatric population [7]. Many treatments have been implicated in the case of idiopathic CTEV, but according to the latest evidence, the gold standard treatment is the Ponseti method [8-9]. However, in syndromic cases, other more invasive surgeries might be required [10].

In order to achieve early diagnosis and treatment of CTEV, public awareness plays a key role [11]. However, a lack of awareness is considered to be a barrier to case management. A few studies have been conducted internationally regarding public awareness of CTEV, and these studies indicate low awareness in the general population [12]. Nevertheless, a few studies involving a small sample size published in Saudi Arabia have revealed that there is low awareness of CTEV [13-14].

## Materials and methods

Study design

This study was a community-based cross-sectional descriptive study conducted in the region of Makkah, Kingdom of Saudi Arabia, 2022.

Study population and sampling methodology

Study participants were drawn from the public who resided in the Makkah region of Saudi Arabia and agreed to participate in the study. In addition, all participants who had not agreed to participate or who were not located in the Makkah region were excluded. Data were collected through a previously validated online questionnaire in published studies [13-14]. It was formulated in Arabic, was completed using Google Documents, and distributed electronically via social media applications. 

The questionnaire covered the following sections: A) The participants' sociodemographic data, including gender, age in years, marital status, and education. B) Level of participants' knowledge and awareness about clubfoot.

Data analysis

The SPSS software Ver. 26 (IBM Inc., Armonk, NY) was used for data entry and analysis. Descriptive statistics such as mean score and standard deviation, as well as frequency and percentages of all independent variables, were used. Responses were scored by frequency and percentage then converted to percentage mean scores, then transformed into qualitative data as mentioned previously. Analytic statistics was applied. The Chi-square test was used for qualitative data while the student’s t-test and analysis of variance (ANOVA) were used for quantitative data. The level of significance is considered at p-value<0.05.

Ethical considerations

Ethical approval was provided by the institutional review board (IRB) of the Directorate of Health Affairs - Taif (No. HAP-02-T-067-2022-07-713). Consent was obtained electronically from all participants after the study aims were explained.

## Results

A total of 1,987 participants were finally enrolled in the current study. Table 1 shows that 979 (49.3%) were males and 1008 (50.7%) were females. With regard to age groups: most of the participants 976 (49.1%) were within the age group of 21-30 years old, 394 (19.8%) of the participants were within the age group of fewer than 20 years old, 247 (12.4%) were within the age group of 31-40 years old, 248 (12.5%) were within the age group of 41-50 years old, and 122 (6.1%) were within the age group of more than or equal to 51 years old. About 1287 (64.8%) of the participants were single, 644 (32.4%) were married, and 56 (2.8%) were divorced. Educational level for most of the participants: 822 (41.4%) were found to be university students, 646 (32.5%) were bachelor's degree holders or above, 392 (19.7%) were with secondary educational level, and 127 (6.4%) were with a diploma. 

**Table 1:** Socio-demographic characteristics of the study respondents (n=1,987).

Variable	Category	Frequency	Percent (%)
Gender	Male	979	49.3
	Female	1008	50.7
Age (years)	≤ 20	394	19.8
	21-30	976	49.1
	31-40	247	12.4
	41-50	248	12.5
	≥ 51	122	6.1
Marital status	Single	1287	64.8
	Married	644	32.4
	Divorced/Widowed	56	2.8
Educational level	Secondary or less	392	19.7
	University student	822	41.4
	Diploma	127	6.4
	Bachelors or above	646	32.5

According to Table 2, there were 831 (41.8%) respondents who had prior knowledge of CTEV. There were 185 (about 9.3%) parents with children who had CTEV, of whom 153 (82%) received information on CTEV, whereas 32 (17.3%) did not receive information on CTEV. With regard to knowledge about risk factors for CTEV; the most reported risk factor was a mispositioned fetus as mentioned by 783 (39.4%) of the participants, 665 (33.5%) reported hereditary and genetic factors, 483 (24.3%) mentioned twins pregnancy, 326 (16.4%) stated intrauterine deficiency of amniotic fluids, 286 (14.4%) reported neurological disorders, 176 (8.9%) mentioned sex of the newborn whereas 215 (10.8%) mentioned other causes, and 715 (36%) do not know. About 655 (33%) of the participants mentioned that the first-line treatment for CTEV is physiotherapy, 384 (19.3%) reported cast as the first-line treatment for clubfoot, and surgery was known as the first-line treatment for about 213 (10.7%) of the participants whereas 735 (37%) do not know. About 724 (36.4%) of the participants agreed that CTEV treatment should be initiated within the first six months of life, 308 (15.5%) mentioned that it should be initiated within the first 6-12 months of age, and 14 (7%) reported that it should be initiated within 1-4 years whereas 815 (41%) do not know. When asked about the percentage of children with CTEV; about 436 (21.9%) mentioned it as 0-20% of children are living with CTEV, 189 (9.5%) stated that the percentage of children with CTEV is from 21% to 40%, 100 (5%) mentioned it as 41%-60%, 39 (2%) stated it as 61%-80%, 39 (2%) reported it as 81%-100%, and 1184 (59.6%) of the participants do not know.

**Table 2:** Awareness and knowledge of CTEV among the population in Makkah region, Saudi Arabia. CTEV, congenital talipes equinovarus *More than one choice can be selected

Variable	Categories	Frequency	Percent (%)
Have you ever heard or read about CTEV?	Yes	831	41.8
	No	1156	58.2
Do you have a CTEV child?	Yes	185	9.3
	No	1802	90.7
Are you informed about CTEV because you have an affected child? (n=185)	Yes	153	82.7
	No	32	17.3
In your opinion, what are the risk factors that lead to CTEV deformity?*	Twin pregnancy	483	24.3
	Sex of the newborn	176	8.9
	Neurological disorders	286	14.4
	Hereditary and genetic reasons	665	33.5
	Mispositioned fetus	783	39.4
	Intrauterine deficient amniotic fluid	326	16.4
	Other	215	10.8
	I do not know	715	36
What is the first-line treatment of CTEV?	Cast	384	19.3
	Physiotherapy	655	33
	Surgery	213	10.7
	I don't know	735	37
When should CTEV treatment be initiated?	Birth to the first 6 months	724	36.4
	First 6-12 months	308	15.5
	1-4 years	140	7
	I do not know	815	41
What is the percentage of children with CTEV	0-20%	436	21.9
	21%-40%	189	9.5
	41%-60%	100	5
	61%-80%	39	2
	81%-100%	39	2
	I don't know	1184	59.6
What are the sources of your information about CTEV?*	Printed media (magazines, newspapers, and books)	173	8.7
	Websites/Internet	537	27
	Television and radio	125	6.3
	Relatives and friends	412	20.7
	Social media	463	23.3
	Affected persons	206	10.4
	Other	902	45.4

The sources of information about CTEV were websites and the internet as stated by 537 (27%) of the participants, social media reported by 463 (23.3%) of the participants as their source of information, 412 (20.7%) mentioned relatives and friends, 20 (10.4%) reported that they got the information from the affected person, 173 (8.7%) stated that printed media as newspapers and magazines as their source of information, 125 (6.3%) mentioned TV and radio, and 902 (45.4%) reported other sources. 

**Figure 1:**
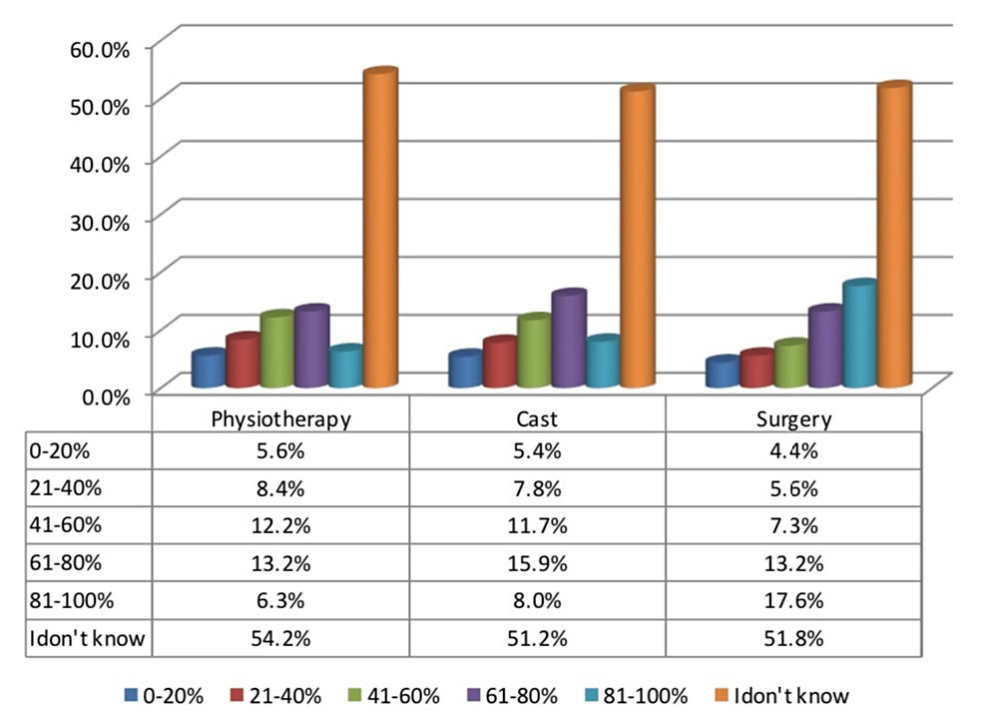
Respondents answered on recovery rates for the various CTEV treatments. CTEV, congenital talipes equinovarus

When asked about recovery rates for the various CTEV treatment options, Figure 1 shows that with regard to physiotherapy, about (13.2%) of the participants mentioned recovery rates of 61%-80%, (12%) of the participants reported recovery rates of 41%-60%, (8.4%) reported recovery rates of 21%-40%, (6.3%) stated recovery rates of 81%-100%, (5.6%) mentioned recovery rates of 0-20%, and about (54.2%) do not know the exact recovery rates after physiotherapy. Concerning knowledge about recovery rates of CTEV after cast, (15.9%) of the participants mentioned recovery rates of 61%-80%, (11.7%) reported recovery rates of 41%-60%, (8%) reported recovery rates of 81%-100%, (7.8%) stated recovery rates of 21%-40%, (5.4%) mentioned recovery rate of 0-20%, and (51.2%) do not know the exact recovery rates. Regarding knowledge about recovery rates after surgery, (17.6%) of the participants reported recovery rates of 81%-100%, (13.2%) mentioned recovery rates of 61%-80%, (7.3%) stated recovery rates of 41%-60%, recovery rates of 21%-40% were reported by 5.6% of the participants, (4.4%) mentioned recovery rates of 0-20%, and (51.8%) do not know about the recovery.

According to Table 3 regarding factors associated with awareness about CTEV, gender was found to be significantly correlated with awareness (p-value = 0.007), with females having a higher awareness level than males. There is a significant correlation between having a child with CTEV and awareness level (p-value=0.001), with participants who have a child with CTEV having a higher awareness level than those without a child with CTEV. Educational level and awareness about clubfoot were found to be significantly associated (p-value = 0.023) with university and secondary educational levels having higher awareness levels compared to others. Knowledge about first-line treatment was found to be significantly associated with clubfoot (p-value ˂0.001) as participants answering cast had higher awareness about first-line treatment (p-value=0.001) compared to those not answering cast. In addition, participants who said the best time for initial treatment of CTEV is from birth to six months tend to have a higher awareness level than others about CTEV (p-value=0.001). Age and marital status were not found to be significantly associated with awareness about CTEV (p-value = 0.115 and 0.830, respectively). 

**Table 3:** Factors associated with awareness about CTEV. CTEV, congenital talipes equinovarus

Variable	Category	Awareness of CTEV		p value
		No	Yes	
		N (%)		
Gender	Male	599 (61.2)	380 (38.8)	0.007
	Female	557 (55.3)	451 (44.7)	
Age (years)	≤ 20	227 (57.6)	167 (42.4)	0.115
	21-30	565 (57.9)	411 (42.1)	
	31-40	131 (53)	116 (47)	
	41-50	161 (64.9)	87 (35.1)	
	≥ 51	72 (59)	50 (41)	
Marital status	Single	743 (57.7)	544 (42.3)	0.830
	Married	379 (58.9)	265 (41.1)	
	Divorced/Widowed	34 (60.7)	22 (39.3)	
Educational level	Secondary or less	214 (54.6)	178 (45.4)	0.023
	University student	463 (56.3)	359 (43.7)	
	Diploma	72 (56.7)	55 (43.3)	
	Bachelors or above	407 (63)	239 (37)	
Do you have a CTEV child?	Yes	23 (12.4)	162 (87.6)	< 0.001
	No	1133 (62.9)	669 (37.1)	
What is the first line treatment of CTEV?	Cast	152 (39.6)	232 (60.4)	< 0.001
	Physiotherapy	351 (53.6)	304 (46.4)	
	Surgery	108 (50.7)	105 (49.3)	
	I do not know	545 (74.1)	190 (25.9)	
When should CTEV treatment be initiated?	Birth to the first 6 months	346 (47.8)	378 (52.2)	< 0.001
	First 6-12 months	163 (52.9)	145 (47.1)	
	1-4 years	70 (50)	70 (50)	
	I do not know	577 (70.8)	238 (29.2)	

## Discussion

Determination and measurement of the exact level of awareness about CTEV among the population is considered to be significant as it might affect the parental decision and preference of management of their child with CTEV and lack of awareness may lead to loss of patience and dissatisfaction about treatment [15]. Less than half (41.8%) of the participants have previously heard about CTEV; this percentage is slightly higher when compared to the parallel study carried out in which only 31.3% of the participants have previously heard about CTEV [13]. About (9.3%) of the participants were having a child with CTEV although the global prevalence was found to be one per thousand as mentioned in the study but this varies across counties [16]. The vast majority (82%) of those who have a child with CTEV received information about CTEV and the rest of them were not educated about CTEV. Concerning knowledge about risk factors for CTEV; the most reported risk factor was a mispositioned fetus as mentioned by (39.4%) of the participants followed by hereditary and genetic factors as reported by (33.5%) of the participants then twins pregnancy which was mentioned by (24.3%) of the participants and intrauterine deficiency of amniotic fluids as stated by (16.4%) of the participants; this was found to be consistent with the findings reported in two studies both of which reported that intrauterine environment as the most noted risk factor for CTEV [3, 17]. 

About one-third (33%) of the participants mentioned that the first-line treatment for CTEV is physiotherapy, nearly one-fifth (19.3%) reported cast as the first-line treatment for CTEV, and surgery was known as the first-line treatment for about (10.7%) of the participants whereas more than one-third (37%) do not know similar findings were reported in the congruent study in which casting was the most reported first-line management followed by surgery [18]. More than one-third (36.4%) of the participants agreed that CTEV treatment should be initiated within the first six months of life, (15.5%) mentioned that it should be initiated within the first 6-12 months of age, and (7%) reported that it should be initiated within 1-4 years whereas less than half (41%) do not know these findings were consistent with the findings from Alam et al. study in which 41.1% of the participants reported it should be managed from birth to 6 months and 37% reported that it should be from 6 to 12 months [12].

The most reported source of information about CTEV was websites and the internet as stated by less than one-third (27%) of the participants followed by social media which was reported by (23.3%) of the participants, then relatives and friends as mentioned by (20.7%) of the participants and others and this was found to be similar to the findings of the congruent study except for that relatives and friends were the most reported source of information about CTEV followed by websites [19]. Physiotherapy was one of the most reported treatment options when asked about recovery rates for CTEV; however, 13.2% of participants reported recovery rates of 61%-80% after physiotherapy, while 54.2% are unaware of the exact recovery rates after physiotherapy.

Concerning factors associated with awareness about CTEV; Gender was found to be significantly associated with awareness about CTEV with females tending to have higher awareness levels than males. Having a child with clubfoot was found to be significantly associated with awareness level with participants having a child with CTEV being found to be having higher awareness levels compared to others. There is a significant correlation between educational level and awareness of CTEV, with university and secondary education levels having higher awareness than other educational levels. Knowledge about first-line treatment was found to be significantly associated with CTEV as participants answering cast had higher awareness about first-line treatment (p-value=0.001) compared to those not answering cast. In addition, participants who said the best time for initial treatment of CTEV is from birth to six months tend to have a higher awareness level than others about CTEV (p-value=0.001). The previously mentioned associations were found to be consistent with the findings of the parallel study in which gender and having an affected child were found to be significantly associated with awareness about CTEV [19]. Age and marital status were not found to be significantly associated with awareness about CTEV.

Study limitations

We are aware of a few study limitations that should be addressed. First, the study collected data using an online questionnaire, which may affect its validity if the answers were researched. The population of the Makkah region of Saudi Arabia is not high enough to represent the population of Saudi Arabia; hence, the results cannot be generalized to the rest of the kingdom.

## Conclusions

In conclusion, the survey revealed that little public awareness exists regarding CTEV. The treatment modalities and net recovery rate of the affected children should be explained to the population in order to raise awareness and improve their perception. Health education sessions should be held for high-risk groups to ensure they understand the nature of the disorder and give hope for the outcome of postnatal treatment. Public awareness can be increased through social media, but only when it is combined with credible sources, such as healthcare professionals.

## References

[1] Gray K, Pacey V, Gibbons P, Little D, Burns J (2014). Interventions for congenital talipes equinovarus (clubfoot). Cochr Datab Syst Rev.

[2] Pavone V, Chisari E, Vescio A, Lucenti L, Sessa G, Testa G (2018). The etiology of idiopathic congenital talipes equinovarus: a systematic review. J Orthop Surg Res.

[3] Siapkara A, Duncan R (2007). Congenital talipes equinovarus: a review of current management. J Bone Joint Surg Br.

[4] Dobbs MB, Gurnett CA (2012). Genetics of clubfoot. J Pediatr Orthop B.

[5] Mason CA, Kirby RS, Sever LE, Langlois PH (2005). Prevalence is the preferred measure of frequency of birth defects. Birth Defects Res A Clin Mol Teratol.

[6] Lochmiller C, Johnston D, Scott A, Risman M, Hecht JT (1998). Genetic epidemiology study of idiopathic talipes equinovarus. Am J Med Genet.

[7] Smythe T, Kuper H, Macleod D, Foster A, Lavy C (2017). Birth prevalence of congenital talipes equinovarus in low- and middle-income countries: a systematic review and meta-analysis. Trop Med Int Health.

[8] Shabtai L, Specht SC, Herzenberg JE (2014). Worldwide spread of the Ponseti method for clubfoot. World J Orthop.

[9] Pavone V, Testa G, Costarella L, Pavone P, Sessa G (20131). Congenital idiopathic talipes equinovarus: an evaluation in infants treated by the Ponseti method. Eur Rev Med Pharmacol Sci.

[10] Hosseinzadeh P, Kelly DM, Zionts LE (2017). Management of the relapsed clubfoot following treatment using the Ponseti method. J Am Acad Orthop Surg.

[11] Rasheed N, Zaidi IH, Rasheed N, Hussain G (2017). Awareness regarding clubfoot in parents. J Pak Orthop Assoc.

[12] Alam Z, Haque MM, Bhuiyan MR, Islam MS, Haque M, Islam AM, Pradhania MS (2015). Assessing knowledge on clubfoot among parents having children with clubfoot deformity. Chattagram Maa-O-Shishu Hosp Med Coll J.

[13] Alsiddiky A, Alrwibaah S, Alqahtani A, Alnujidi A, Alhomaidhi A, Almasoud A, Alatassi R (2019). Assessing public awareness of clubfoot and knowledge about the importance of early childhood treatment: a cross-sectional survey. BMC Pediatr.

[14] Alfaya FF, Alqahtani YM, Almutairi HA (2020). Awareness level of general population regarding club foot in Aseer region, Southern of Saudi Arabia. Middle East J Fam Med.

[15] McCabe KM (2002). Factors that predict premature termination among Mexican-American children in outpatient psychotherapy. J Child Fam Stud.

[16] Ansar A, Rahman AE, Romero L (2018). Systematic review and meta-analysis of global birth prevalence of clubfoot: a study protocol. BMJ Open.

[17] Dietz F (2002). The genetics of idiopathic clubfoot. Clin Orthop Related.

[18] Iqbal MS, Dubey R, Thakur K, Katiyar S, Prasad M (2021). Assessment of awareness and barriers to clubfoot treatment in the Indian scenario. J Fam Med Prim Care.

[19] Almogbil I, B Albaker A, Alrabai H (2021). The level of public awareness about clubfoot in the Al-Qassim region and importance of early childhood intervention: a cross-sectional study. Pak J Med Health Sci.

